# The Effect of Graphene Oxide Deposited on Titanium Surface on Structural, Corrosion, and Biological Properties

**DOI:** 10.3390/ma18235372

**Published:** 2025-11-28

**Authors:** Kamila Narojczyk, Barbara Nasiłowska, Agata Lange, Marta Kutwin, Sławomir Jaworski, Łukasz Krzowski, Wiktoria Kasprzycka, Piotr Olejnik, Maciej Chrunik, Aneta Bombalska, Zdzisław Bogdanowicz

**Affiliations:** 1 Doctoral School, Military University of Technology, gen. S. Kaliskiego 2, 00-908 Warsaw, Poland; kamila.narojczyk@student.wat.edu.pl; 2 Institute of Optoelectronics, Military University of Technology, gen. S. Kaliskiego 2, 00-908 Warsaw, Poland; 3 Department of Nanobiotechnology, Institute of Biology, Warsaw University of Life Sciences, Ciszewskiego 8, 02-786 Warsaw, Poland; 4 Department of Physics and Biophysics, Warsaw University of Life Sciences (SGGW), 159 Nowoursynowska Street, 02-776 Warsaw, Poland; 5 Institute of Applied Physics, Military University of Technology, gen. S. Kaliskiego 2, 00-908 Warsaw, Poland; 6 Faculty of Mechanical Engineering, Military University of Technology, gen. S. Kaliskiego 2, 00-908 Warsaw, Poland

**Keywords:** graphene oxide, titanium, implants, human skin melanoma cell line Hs 895.T, human skin fibroblast cell line Hs 895.Sk

## Abstract

The article presents the results of structural, corrosion, microbiological, biological, and genotoxicity studies on the effect of graphene oxide deposited on a flat titanium foil surface, intended for use, in general, implantology and other medical applications. The methodology of graphene oxide (GO) deposition involved a surface cleaning process combined with RF plasma activation, followed by the application of a thin layer of dispersed aqueous GO suspension using a spin coater. The graphene oxide layer was uniformly deposited on the surface, which was confirmed by SEM imaging. Corrosion studies were carried out in an electrochemical cell filled with a buffered solution prepared to mimic the composition of physiological intracellular fluids. It was demonstrated that the deposition of graphene oxide on the titanium surface limited the access of electrolyte and oxygen. Surface activation and deposition of the aqueous graphene oxide suspension contributed to improved adhesion, condition, growth, and proliferation of fibroblast cell lines Hs 895.T and Hs 895.Sk. The inhibition zone analysis revealed a bacteriostatic effect against *Pseudomonas aeruginosa* and *Staphylococcus aureus*. Moreover, no genotoxicity changes were observed.

## 1. Introduction

Graphene oxide (GO), due to its unique properties such as a large specific surface area, the presence of oxygen-containing functional groups, good solubility, and colloidal stability, has found wide application in bioengineering [1,2]. Research presented by Lee et al. [3] demonstrated that GO can stimulate the growth of stem and neural cells, also improving their adhesion. Scaffolds made of GO create a suitable environment for chondrocyte growth, promoting cartilage regeneration and joint repair [4]. GO can stimulate osteogenic differentiation, supporting bone formation, which makes it a promising material for bone tissue regeneration [3]. It is also used as an innovative component of biofilaments for 3D tissue printing, enabling the creation of advanced biomedical structures that support tissue regeneration [5]. Due to its good solubility in water and the presence of reactive functional groups, GO can effectively absorb enzymes, which is useful for cell imaging and drug delivery applications [6].

Ryu et al. [7] showed that both graphene (G) and graphene oxide (GO) are biocompatible materials that provide favorable conditions for effective cell proliferation. Graphene is characterized by certain unique properties, such as high electrical conductivity and good absorption of proteins and other low-molecular-weight compounds. This has significant implications for cell development, as cells interacting with their surrounding structures secrete substances that influence the proliferation process [8].

Wong Cheng Lee et al. [3] conducted studies on MSCs (mesenchymal stem cells), which have the ability to differentiate into various cell types, primarily osteocytes, chondrocytes, and adipocytes. MSCs derived from human bone marrow were cultured on three substrates: graphene (G), graphene oxide (GO), and PDMS (polydimethylsiloxane)—a silicone biocompatible membrane. The conducted research [3] showed significant differences in cell morphology. Initially, for MSCs cultured on G and GO substrates, numerous filopodia and protrusions were observed, allowing for intercellular interactions. These structures were not visible in cells cultured on PDMS matrices, where cells appeared rounder and less adherent to the surface. The number of cells grown on G and GO substrates was significantly higher compared to the control.

For hASC—human adipose-derived mesenchymal stem cells—the team of Kim et al. [8] reported similar findings. GO films provided a favorable environment for hASC development, adhesion, proliferation, and differentiation.

Aryaei et al. [9] evaluated the biocompatibility of graphene layers using silicon substrates. In the case of silicon plates coated with GO layers, an increased number of “attached” osteoblasts was observed (326 ± 60 cells) compared to the uncoated substrate (280 ± 70). The cells on the uncoated silicon plate exhibited undeveloped morphology with a rounded shape, while those on the GO-coated plates showed an elongated morphology and numerous filopodia. Moreover, the authors [9] reported that no cytotoxic effects were observed in either group. The results suggest that applying graphene layers to bone implants may enhance osteoblast adhesion and growth.

Liu et al. [10] conducted studies confirming the antibacterial activity of GO against the Escherichia coli strain. These studies showed that the activity of GO depends on incubation time and concentration (the percentage of dead bacterial cells increased proportionally with both parameters). Disruption of cell membrane structure and function (membrane stress) and excessive accumulation of reactive oxygen species (oxidative stress) were observed. The antibacterial effects of graphene oxide against various bacterial strains have also been confirmed in other scientific publications [11,12,13].

The antimicrobial efficacy of GO against both Gram-positive and Gram-negative bacteria has been reported as well. In a review by Romiszewska et al. [14], the effect of graphene oxide on bacterial growth was analyzed. Significant inhibition was observed, although the effectiveness of GO depended on physical bacterial characteristics such as shape and size.

The use of GO-enriched tissue scaffolds has been shown to improve wound healing and reduce bacterial infections [15]. Necolau et al. [16] presented studies on chitosan hydrogels containing graphene oxide, which were found to enhance tissue regeneration and exhibit antibacterial efficacy. Similarly, Ferrari et al. [17] demonstrated that the use of graphene as a substrate supporting cell growth and tissue regeneration showed no signs of toxicity.

An important issue in the functional performance of implants is their corrosion resistance when exposed to body fluids. In clinical studies conducted by Bao [18], where titanium elements were immersed in patients’ synovial fluids, the stable open circuit potential (OCP) ranged between −151 and −529 mV vs. Ag/AgCl. In typical corrosion experiments by Boraei [19], the OCP of Ti alloys shifted toward more positive potentials. According to experimental data reported by [20], surface coverage/passivation of TiO_2_ shifts the OCP to approximately 103 mV.

Modification of the surface layer of titanium implants through micro-milling [21], UV radiation treatment [22], and etching [23] can influence fibroblast viability, proliferation, adhesion, and activation. Baraka et al. [24] presented the results of studies on the attachment of human gingival fibroblasts (HGF) of soft tissue to titanium dioxide nanotubes (TNTs) compared with commercially pure titanium (cp-Ti) and its alloys, in in vitro research. A significant increase in cell proliferation on TNT surfaces compared to cp-Ti on day 7 in three studies and on day 14 in one study was observed. A significant increase in type I collagen protein expression on TNTs compared to cp-Ti on day 6 in one study and on day 7 in two studies was also reported.

Previous studies on GO-modified titanium have focused mainly on complex hybrid systems, such as GO incorporated into anodically formed TiO_2_ nanotube arrays [25] or GO/gelatin composite coatings applied to titanium substrates [26]. Other work has examined GO deposited on titanate nanowire scaffolds rather than on metallic titanium surfaces [27]. These approaches differ fundamentally from the system investigated in the present study. Additionally, there are limited data that explore the GO layer deposited directly onto unalloyed, flat titanium foil, without nanotubular or nanowire oxide structures or alloying components. Moreover, earlier studies typically focused on a single biological parameter—such as osteoblast adhesion or antibacterial behavior—whereas none provided a comprehensive evaluation encompassing cytocompatibility, morphology, bacterial response, electrochemical stability, and genotoxicity. Given these limitations, there is a clear need to examine how a uniform GO coating on unalloyed titanium foil influences cellular and microbiological responses under conditions relevant to medical implants.

A review of the literature concerning the application of graphene oxide in biomedical engineering highlights its antibacterial and biocompatible properties, as well as its ability to stimulate cell growth and proliferation. This indicates the potential of GO as an innovative biomaterial supporting tissue engineering and the development of modern regenerative therapies. However, there is a lack of studies investigating the effect of graphene oxide deposited on titanium surfaces—to general implantology and medical applications—on human fibroblast cells, including healthy fibroblasts (Hs 895.Sk, CRL–7636) and cancerous fibroblasts (Hs 895.T, CRL–7637). This issue is particularly important in oncology patients requiring surgical interventions involving implant placement.

The aim of the biological part of this study was to comprehensively assess the cytocompatibility of titanium foils modified with graphene oxide (Ti + GO) in contact with human skin cells and representative bacterial strains. The research included quantitative evaluation of viability, adhesion, and proliferation of healthy skin fibroblasts (Hs 895.Sk) and melanoma-derived fibroblast-like cells (Hs 895.T) cultured on Ti and Ti + GO, verification of potential genotoxic effects using the cytokinesis-block micronucleus assay, characterization of cell morphology and cell–substrate interactions using SEM, and evaluation of antibacterial responses against *Staphylococcus aureus* and *Pseudomonas aeruginosa* via inhibition zone tests, viability assays, and SEM imaging.

It was hypothesized that titanium surface functionalization with graphene oxide would enhance cytocompatibility and cell adhesion while maintaining normal morphology and lack of genotoxicity, as well as exhibit a bacteriostatic effect compared to pure titanium, confirming the suitability of Ti + GO surfaces for implantology applications.

## 2. Materials and Methods

The studies on graphene oxide deposited on the titanium surface were carried out on samples with the following designations:

- Ti—titanium foil in the as-received state;
- Ti + GO—titanium foil with a deposited layer of graphene oxide.

Model surgical implants were made from titanium foil (Ti) with a purity of 99.6% Ti according to the material data sheet (GoodFellow, Huntingdon, UK) [28]. The samples used in the study measured 5 × 5 × 0.025 mm. All experiments presented in this work were performed in 3–5 repetitions.

The aqueous dispersion of graphene oxide was purchased from the Institute of Electronic Materials Technology (ITME, Warsaw, Poland) [29].

The first stage of the study involved coating the titanium foil with a layer of graphene oxide (Ti + GO). For this purpose, the samples were cleaned with ethyl alcohol, then the surface was activated and cleaned using RF plasma at a power of 100 W for approximately 15 min (Plasma Prep III, Garfield Ave, Westchester, PA, USA). This procedure was applied based on literature reports. Publications [30,31,32] demonstrated that plasma treatment of steel surfaces improves their hydrophilicity, thereby enhancing the distribution of aqueous suspensions containing graphene oxide flakes.

After plasma treatment, the samples were removed from the chamber and placed on Petri dishes. A dispersed suspension of graphene oxide (4.5 g/L) (Institute of Electronic Materials Technology, ITME, Warsaw, Poland) was applied to the samples. To remove excess suspension, the spin coating technique was used (POLOS Spin150i—NPP, SPS-Europe B.V., Putten, The Netherlands) with a set rotation speed of 3000 rpm. Additionally, to evaporate excess water, the samples were placed in a Vacucell 22 vacuum oven (MMM Muenchener Medizin Mechanik Polska, Warsaw, Poland).

### 2.1. Structural Analysis 

The contact angle was measured using an optical microscope (6000 VHX, Keyence Corporation, Osaka, Japan). In the wettability tests, individual suspensions were collected using a syringe dispenser. Then, drops of the aqueous graphene oxide suspension, with a volume of approximately 3 µL, were released onto the surface from a fixed height of 5 mm. Five measurements were performed, from which the mean value and standard deviation were calculated.

Imaging of the dispersed graphene oxide suspension was performed using a STEM detector integrated with a Quanta 250 FEG microscope (FEI, Hillsboro, OR, USA). Surface topography studies of Ti and Ti + GO samples were carried out using a CBS detector, also available in the Quanta 250 FEG scanning electron microscope. The applied accelerating voltage was 10 kV with a spot size of 3. For each sample, images were taken at magnifications of 1000× (CBS), 10,000× (CBS), and 100,000× (STEM).

For STEM analysis, copper grids were used, onto which the aqueous dispersion of graphene oxide was applied using centrifugal force (3000 rpm).

To identify carbonyl, carboxyl, and hydroxyl groups, as well as the carbon chain present on the sample surfaces after GO deposition, the samples were analyzed using a Fourier-transform infrared spectrometer (FTIR) (Nicolet IS50, Thermo Fisher Scientific, Waltham, MA, USA) equipped with an ATR (attenuated total reflectance) module. The analyses were conducted in the range of 400–4000 cm^−1^, with a resolution of 4 cm^−1^ and 64 scans applied. Three measurements were performed for each side of the sample.

The studied (Ti and Ti + GO) samples were examined by means of the X-ray diffraction using BRUKER D8 Discover diffractometer (Bruker AXS SE, Karlsruhe, Germany), equipped with CuKα radiator (λKα1 = 1.54056 Å, λKα2 = 1.54443 Å, Siemens KFL CU 2 K, 40 kV voltage, and 40 mA current in operating mode), Göbel FGM2 mirror and 1D LYNXEYE detector (no monochromator) and Bragg–Brentano geometry. Each measurement was performed with at least a 0.015° step size and an acquisition time of 2 s per step. An Anton Paar HTK-1200 N (Anton Paar GmbH, Graz, Austria) temperature chamber, was applied to stabilize the temperature at RT (around 293 K). Each measurement was reduced by the recorded background as well as the λKα2 component. Rietveld refinement and precise calculations of entire diffraction profiles, along with refined unit cell parameters, were performed using the FullProf Suite (Ver. January-2021). Phase analysis was performed using Match! Ver. 4.2 application, provided by Crystal Impact, with support of the COD database [33].

The surface roughness was measured using a Mitutoyo SJ–201 device (Mitutoyo Corporation, Takatsu, Kawasaki, Japan). The Ra and Rz parameters were determined, measurements were repeated five times, and then the arithmetic mean and standard deviation were calculated.

### 2.2. Corrosion Studies

Electrochemical corrosion studies of Ti and Ti + GO samples were carried out using a Metrohm Autolab PGSTAT 101 potentiostat controlled by NOVA 2.1 software. The measurement system consisted of a glass electrochemical cell equipped with a three-electrode setup, where a platinum rod (Ø = 1 mm) served as the auxiliary electrode, Ag/AgCl (3 mol/L KCl) was used as the reference electrode, and the working electrode was the tested Ti or Ti + GO sample.

During each measurement, the electrochemical cell was filled with a buffered solution formulated to simulate the composition of physiological intracellular fluids. For both titanium and graphene oxide–coated titanium samples, long-term open circuit potential (OCP) measurements were first recorded, followed by the acquisition of potentiodynamic polarization curves.

### 2.3. Biological Studies

#### 2.3.1. In Vitro Culture of Human Skin Melanoma Cell Line Hs 895.T and Human Skin Fibroblast Line Hs 895.Sk

The Hs 895.Sk American Type Culture Collection (ATCC), normal skin fibroblast cell line, and Hs 895.T (ATCC©), melanoma cell line, were grown in Dulbecco’s Modified Eagle’s Medium (ATCC©) supplemented with 10% fetal bovine serum (Gibco^®^, Grand Island, NY, USA) and a mixture of antibiotics/antimycotic (Lonza Group AG, Basel, Switzerland): penicillin (10 U/mL) with streptomycin (10 µg/mL). Cell cultures were maintained in an incubator (HERAcell VIOS 160i, Thermo Scientific, Waltham, MA, USA) at 37 °C, 90% humidity, and in an incubator, atmosphere containing 5% CO2 (HERAcell VIOS 160i, Thermo Scientific).

The titanium foils, in their native state and with the GO layer deposited, were exposed to 70% ethyl alcohol for 15 min and then to UV-C radiation for 30 min to sterilize the surface of the samples. Then, the foil fragments were placed in a 24-well plate, and 1 mL of a cell suspension with a density of 104 cells/mL was added. The multi-well plate prepared in this manner was incubated for 24 and 48 h at 37 °C with 95% humidity and 5% CO_2_.

#### 2.3.2. Cell Viability of Human Skin Melanoma Cell Line Hs 895.T and Human Skin Fibroblast Line Hs 895.Sk

The viability of human skin melanoma cells (Hs 895.T) and human skin fibroblasts (Hs 895.Sk) after 24–48 h of incubation on titanium foils coated with a graphene oxide layer was assessed using the LIVE/DEAD™ Viability/Cytotoxicity Kit (Invitrogen, Waltham, MA, USA), in accordance with the manufacturer’s instructions. After incubation, the cells were observed using a LSM 700 Axio Observer.Z1 confocal microscope (Carl Zeiss Microscopy, Jena, Germany), following prior maintenance of the samples at 37 °C and protection from light exposure.

#### 2.3.3. Morphology of Human Skin Melanoma Cell Line Hs 895.T and Human Skin Fibroblast Line Hs 895.Sk

The morphology of the cells was visualized using a scanning electron microscope (SEM). For this purpose, cells proliferating on the surface of titanium foils (Ti) and titanium foils with a deposited graphene oxide layer (Ti + GO) were fixed with a mixture of 4% paraformaldehyde (Sigma-Aldrich, Burlington, MA, USA) and 0.4% glutaraldehyde (Sigma-Aldrich, Burlington, MA, USA). The cells were then contrasted in a 1% osmium tetroxide (OsO_4_) solution (Sigma-Aldrich, Burlington, MA, USA) at 4 °C, and dehydrated through a graded series of ethanol (POCH, Gliwice, Poland) and acetone (POCH, Gliwice, Poland) solutions ranging from 30% to 100%. After dehydration, the samples were dried at the critical point using a Leica EM CPD300 (Leica Microsystems GmbH, Wetzlar, Germany) and then coated with a thin layer of gold using a sputter coater (Leica EM ACE200, Leica Microsystems GmbH, Wetzlar, Germany).

#### 2.3.4. Analysis of Bacterial Inhibition Zones

Analysis of bacterial growth and its inhibition after exposure to cement mortar was prepared with two bacterial strains: *Staphylococcus aureus* (ATCC 25923) and *Pseudomonas aeruginosa* (ATCC 27853), which were obtained from the American Type Culture Collection (ATCC) and they were maintained in 20% (*v*/*v*) glycerol at −20 °C. On the surface of solidified Mueller–Hinton agar prepared in Petri dishes (Ø90 mm), the spread plate method of bacterial suspension (0.5 on the McFarland scale) was performed, and then the samples of cement mortar (10 × 10 mm) were placed on the surface. The plates were incubated in standard conditions (24 h, 37 °C). After incubation, the inhibition zones were measured and captured with an Azure C400 (Azure Biosystem, Dublin, CA, USA). The procedure of preparing samples for this analysis was described in [34,35]. Thereafter, the Ti and Ti + GO samples were removed and prepared for scanning electron microscopy

Due to the limitations that may occur in the method determining the growth inhibition zone, a viability analysis was also performed using the PrestoBlue test (ThermoFisher, Waltham, MA, USA). For this purpose, Ti and Ti + GO samples were placed in Mueller–Hinton broth, to which a 10 µL suspension of bacteria (0.5 on the McFarland scale) was added, and the whole mixture was incubated for 24 h at 37 °C. To the liquid culture, 10 µL of PrestoBlue was added to each well (the sample contained a positive control, i.e., medium with added bacteria, without the test factor, and a negative control, i.e., the medium alone). The whole was incubated for 30 min, and the absorbance was measured at a wavelength of 570 nm and a reference of 600 nm. The results were calculated as the average absorbance (test group-negative control/positive control-negative control)*100%. Each test was performed in at least 3 replicates.

#### 2.3.5. Cell Morphology of Human Skin Melanoma Cell Line Hs 895.T and Human Skin Fibroblast Line Hs 895.Sk

The study was conducted using fibroblast cultures with an average density of 2–3 × 10^5^ cells/mL of medium. The cells were exposed to graphene oxide flakes after 24 h of incubation. Incubation was carried out horizontally in an incubator at 37 °C with 5% CO_2_. After 23 h of culture, 1 µL of cytochalasin B (6 µg/mL) was added and gently mixed, and after another 24 h, the culture was collected. The cells were washed twice in PBS solution.

The next step was to terminate the culture and fix it using a methanol/acetic acid/Ringer’s solution mixture (4/1/5, 4 °C), followed by staining the cells with DAPI Fluoroshield™ with DAPI. The previously prepared fixative was kept at 4 °C for approximately 30 min before use. The culture was mixed and centrifuged for 8 min at 1000 rpm. The supernatant was discarded, leaving about 0.5 cm of medium above the cell pellet. The pellet was vortexed, and during the process, 5 mL of fixative was slowly added. The prepared solution was stored at 4 °C. The cells were then centrifuged again for 8 min at 1000 rpm. The pellet was vortexed, and 5 mL of methanol/acetic acid fixative (4/1, 4 °C) was slowly added. The pellet was vortexed again, and 40 µL of the suspension was transferred onto a microscope slide, fully covering its surface.

Cell density was assessed under a ZEISS Imager Z2 microscope (Carl Zeiss AG, Oberkochen, Germany) equipped with a UV attachment.

## 3. Results

### 3.1. Structural Studies

**Figure 1:**
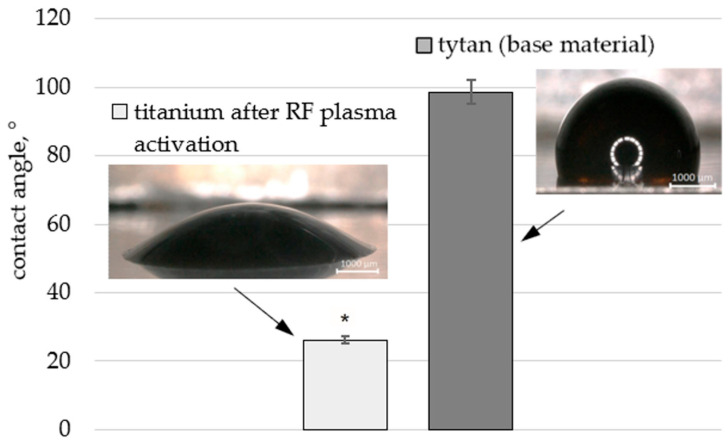
Shape of a dropped aqueous dispersed graphene oxide suspension (4.5 g/L) droplet with a volume of approximately 3 µL on the surface of titanium foil before and after RF plasma activation. Statistical analysis of the obtained results was carried out using ANOVA (*—statistically significant result).

Figure 1 presents the results of the contact angle measurements before and after plasma activation for the titanium foil (Ti) and the foil coated with a dispersed graphene oxide suspension (Ti + GO). A significant increase in surface wettability after exposure to low-temperature RF plasma was observed, amounting to approximately 73.43%. This is important due to the easier distribution of graphene oxide flakes contained in the dispersed suspension across the surface.

**Figure 2:**
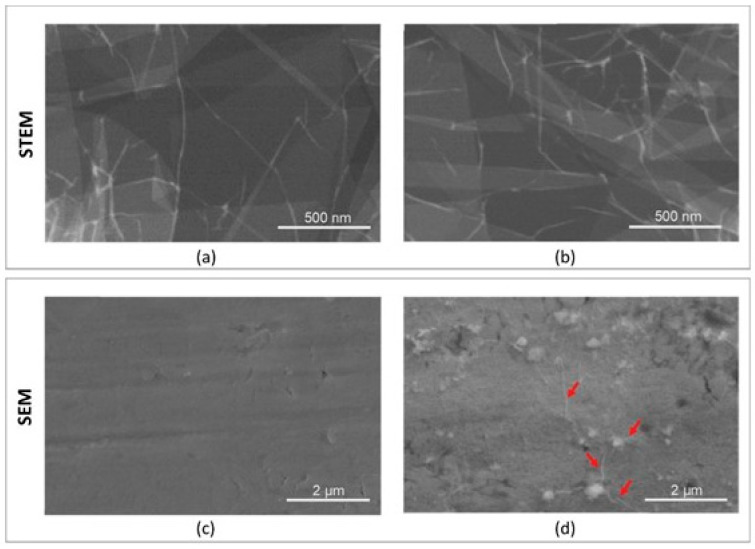
Structure of GO flakes (**a**,**b**) and surface topography of Ti (**c**) and Ti + GO (**d**), obtained using a Quanta 250 FEG microscope at 50,000× magnification.

The use of Scanning Transmission Electron Microscopy (STEM) enabled a qualitative assessment of the GO flakes (Figure 2a,b). Layered arrangements of GO sheets are clearly visible. The size of individual GO flakes ranged from 5 to 45 µm. The analysis did not reveal any contamination in the form of micro- or nanoparticles. A uniform deposition of a thin GO layer was observed, visible only due to the characteristic folds of the flakes and their boundaries (arrows in Figure 2d).

**Figure 3:**
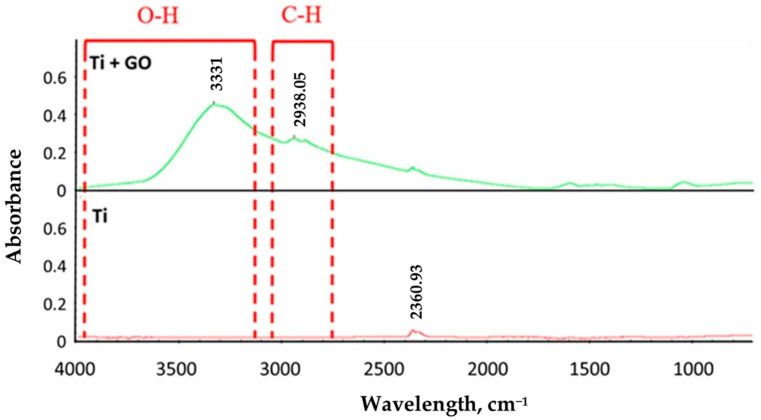
FTIR spectrum of titanium foil (99.6% Ti) in the as-received state (Ti) and titanium foil with a deposited graphene oxide layer (Ti + GO).

Fourier-transform infrared (FTIR) spectroscopy was performed for Ti and Ti + GO samples. Characteristic peaks were observed only for the samples with the deposited GO layer, which is typical for this structure. The peaks at 3331–3361 cm^−1^ correspond to the stretching vibrations of hydroxyl (O–H) groups, while those in the range of 2888–2943 cm^−1^ are characteristic of aliphatic C–H bond. Moreover, small peaks were also observed at 1630 cm^−1^ and 1050 cm^−1^, corresponding to C–O–C bonds, respectively (Figure 3).

**Figure 4:**
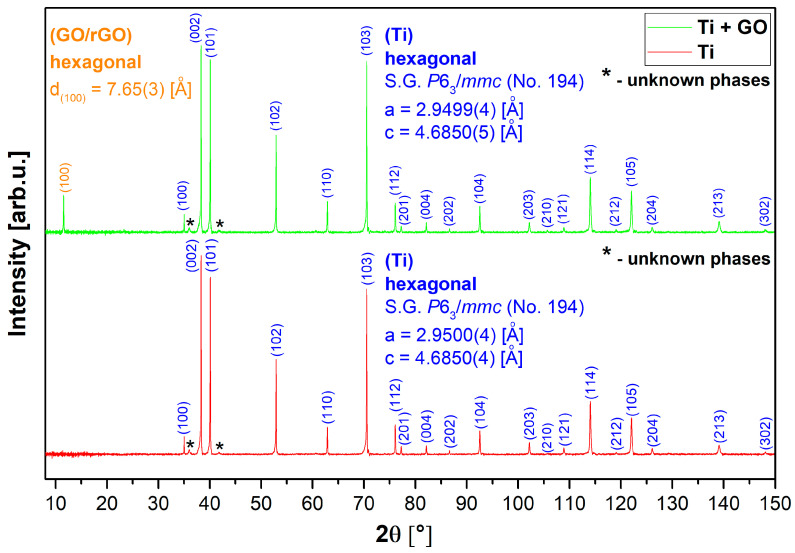
Diffraction patterns of Ti and Ti + GO samples, along with the detected crystallographic planes and their corresponding (hkl) indices and unit cell parameters. Blue font color refers to the titanium phase, orange one to graphene oxide/reduced graphene oxide.

The results of XRD measurements and phase analysis of Ti and Ti + GO samples are presented in Figure 4.

Phase analysis showed that the base substrate for each sample measured was metallic titanium with a hexagonal structure (close-packed, hcp). Detailed parameters of this structure are included in Figure 4. The diffraction reflections recorded from this phase are in very good agreement with the pattern from Wyckoff et al. [36] and the appropriate template card from the COD database (no. [96-900-8518]). However, for each sample tested, a residual signal of an unidentified phase (or phases) was recorded in the form of two very weak reflections (marked as (*)) in the angular range of 35–45°. However, the presence of titanium oxides (TiO_2_) and all its polymorphic variants has been excluded. The refined unit cell parameters for the titanium substrate in both examined samples were almost identical and with similar uncertainties, which allows us to conclude that the applied GO coating did not affect the structure of the substrate. The aforementioned coating gave a single, distinct Bragg reflex above 11°, in a position slightly higher than typical GO coatings. This difference may result from the possibility of partial reduction in GO to rGO at elevated temperature at the preparation stage, which results in a shortening of the interplane distance (100), and this effect depends on the degree of reduction itself [37,38,39]. However, the precision of finding this distance is limited to the factor of ∆d = d(∆λ/λ + ctgθ∙∆θ) and its own θ position, giving uncertainty of the 0.01 Å order of magnitude. Higher diffraction orders from GO/rGO basic plane ((200), (300), etc.) were not recorded due to the strong diffraction signal coming from titanium. The titanium substrate itself showed a good level of crystallinity.

**Figure 5:**
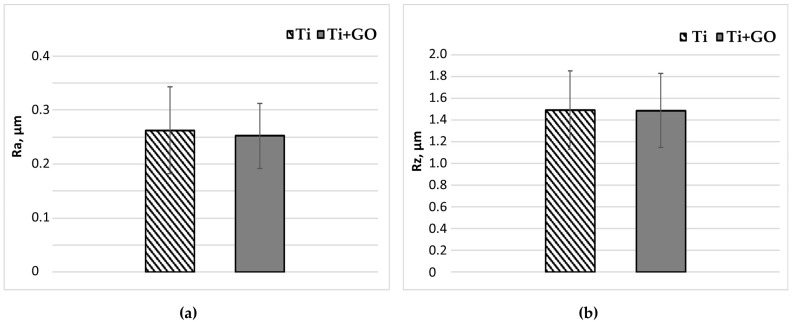
Arithmetic average roughness—Ra (**a**), average maximum height of the roughness profile—Rz (**b**) (mean from five sampling lengths) for Ti and Ti + GO. Statistical analysis of the obtained results was carried out using ANOVA.

Deposition of the top layer on the low-temperature RF plasma-activated titanium surface resulted in a reduction in the Ra and Rz roughness parameters by 1.04% and 0.99%, respectively (Figure 5). Ra—arithmetic average roughness, Rz—average maximum height of the roughness profile (mean from five sampling lengths).

### 3.2. Electrochemical Corrosion

**Figure 6:**
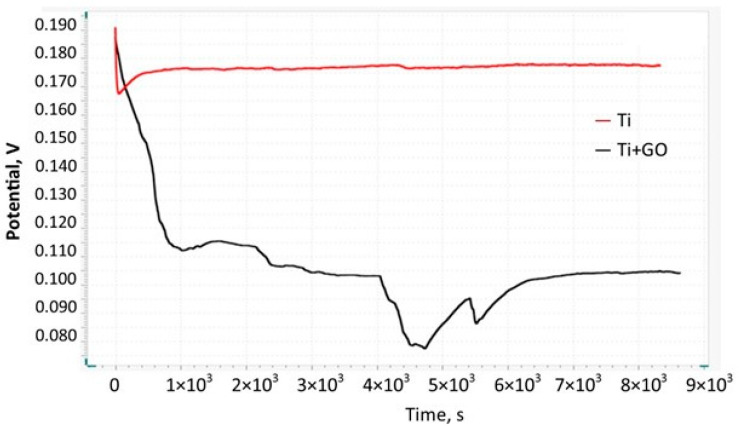
Comparison of long-term OCP curves recorded in a medium simulating rat physiological fluids for Ti and Ti + GO.

Equilibrium-state open circuit potential (OCP) measurements versus the reference electrode were used to assess the potential stability of the Ti and Ti + GO over a 24 h period. Figure 6 presents a comparison of OCP curves for an unmodified Ti and Ti + GO. It should be emphasized that the OCP value is influenced by numerous factors, such as sample geometry, electrolyte composition, and particularly the presence of proteins or other bioorganic species, as well as temperature.

According to Figure 6, the OCP for the rectangular Ti with a working area of 1.13 cm^2^ stabilizes gradually. Initially, in the range from 8000 s to 40,000 s, it stabilizes at approximately 0.116 V, and then decreases to 0.108 V. After approx. 11 h, a multi-stage decrease is observed, reaching a minimum of 0.08 V. Final stabilization occurs after 65,000 s, which allows the OCP value to be set at 0.104 V. One of the advantages of titanium materials is their ability to spontaneous passivation with a TiO_2_ layer, which is observed throughout the entire 24 h experiment. Fluctuations associated with potential drop may be caused by uneven metal surface topography, which directly affects the non-uniform thickness of the passivation layer. In addition, the presence of structurally diverse microdefects may initiate local surface pH changes, adsorption and accumulation of anions present in electrolytes, as well as a decrease in oxygen concentration, which negatively affects the homogeneity and electrochemical stability of the formed protective layer. In the case of titanium coated with graphene oxide, we can see that the application of a thin GO layer limits the access of electrolyte and oxygen to the metal surface. The red curve shows that the electrochemical potential of the Ti + GO composite in the range of 0.175–0.178 V stabilizes very quickly and remains nearly unchanged over time. The higher OCP value of 0.1774 V relative to bare Ti indicates lower surface activity of the material, minimizing the risk of corrosive redox processes and providing improved electrochemical properties, which—when considered in implant applications—positively affect mechanical stability and biocompatibility.

**Figure 7:**
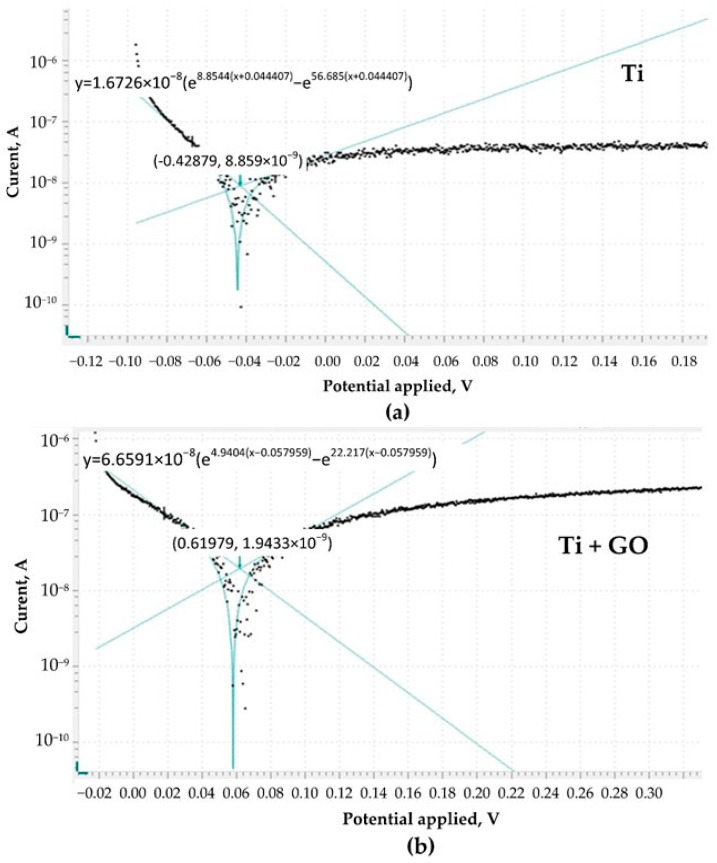
Corrosion rate analysis for plots of Ti (**a**) and Ti + GO (**b**).

Potentiodynamic polarization curves were additionally recorded for both Ti and Ti + GO samples (Figure 7). The measurements were conducted under identical conditions for three plates of each material, using controlled cathodic–anodic polarization in a potential range of ±200 mV relative to the previously determined OCP. The obtained curves exhibit both repeatability and stability. Polarization curves enabled simultaneous analysis of the corrosion potential (Ecorr) and corrosion current density (jcorr), thereby providing complementary information regarding the corrosion tendency and the kinetics of the corrosion process.

These parameters were estimated by fitting a model through linear extrapolation of the cathodic and anodic of the Tafel plots, as shown in Figure 7a,b. It can be seen that the polarization curve representing Ti + GO is shifted toward more positive potentials compared to unmodified Ti, while the corrosion current density for Ti + GO increases. Such a shift in the corrosion potential may indicate enhanced corrosion protection; however, the increase in corrosion current may be related to the development of the electroactive surface due to the graphene oxide coating, which is characterized by high porosity and a large specific surface area. As a material with poor conductivity, GO should have little impact on the recorded current value. The higher corrosion current value may also be attributed to non-uniform coverage of the Ti surface by GO and the presence of microdefects where corrosion is locally accelerated. The kinetic parameters of the potentiodynamic polarization curves are summarized in Table 1.

### 3.3. Cell Viability Assessment

In the viability studies of the Hs 895.Sk and Hs 895.T cell lines using the LIVE/DEAD™ Viability/Cytotoxicity Kit, the number of live cells (green color) and dead cells or those with damaged cell membranes (red color) was evaluated. Images of human skin fibroblasts (Hs 895.Sk) and melanoma cells (Hs 895.T) were obtained using confocal microscopy (LSM 700 Axio Observer.Z1, Carl Zeiss Microscopy, Jena, Germany).

**Figure 8:**
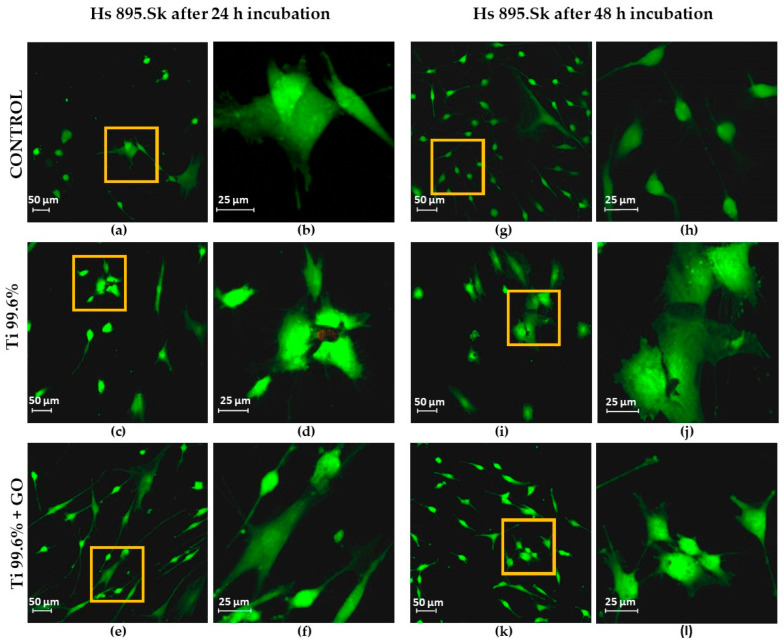
Assessment of the viability of healthy fibroblast cells of the Hs 895.Sk line after 24 h of incubation: control (**a**,**b**), cultured on Ti (**c**,**d**) and Ti + GO (**e**,**f**); and after 48 h of incubation: control (**g**,**h**), cultured on Ti (**i**,**j**) and Ti + GO (**k**,**l**) (LIVE/DEAD™ Viability/Cytotoxicity Kit stain), yellow squares show the magnified area.

**Figure 9:**
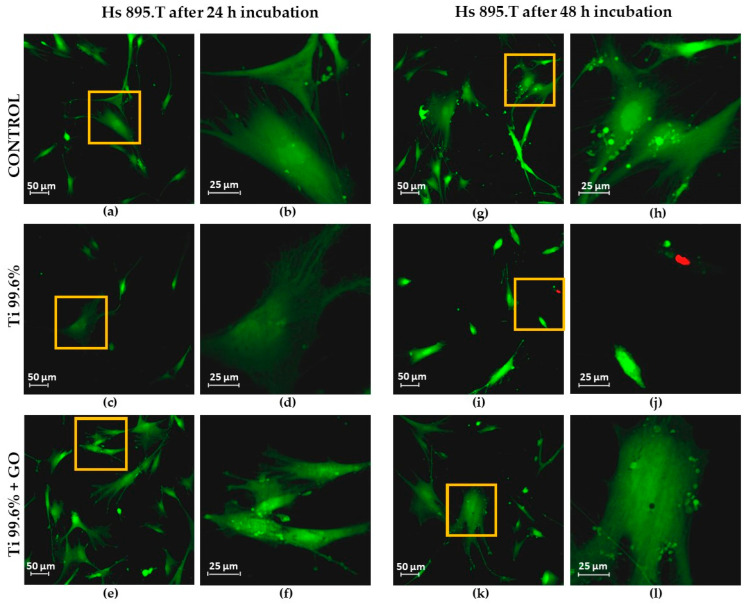
Assessment of the viability of cancerous fibroblast cells of the Hs 895.T line after 24 h of incubation: control (**a**,**b**), cultured on Ti (**c**,**d**) and Ti + GO (**e**,**f**); and after 48 h of incubation: control (**g**,**h**), cultured on Ti (**i**,**j**) and Ti + GO (**k**,**l**) (LIVE/DEAD™ Viability/Cytotoxicity Kit stain), yellow squares show the magnified area.

Fibroblasts were observed after 24 h of incubation (Figure 8a,b,e,f,i,j) and 48 h of incubation (Figure 8c,d,g,h,k,l) at 37 °C in an atmosphere containing 5% CO_2_. Similarly, melanoma cells exhibiting fibroblast-like morphology were observed after 24 h (Figure 9a,b,e,f,i,j) and 48 h of incubation (Figure 9c,d,g,h,k,l) under identical culture conditions.

Figure 8e–h and Figure 9e–h show cells cultured on titanium substrates (Ti), while those grown on titanium coated with graphene oxide (Ti + GO) are presented in Figure 8i–l and Figure 9i–l. Control samples included Hs 895.Sk (Figure 8a–d) and Hs 895.T (Figure 9a–d) cells that were not exposed to Ti or Ti + GO.

**Figure 10:**
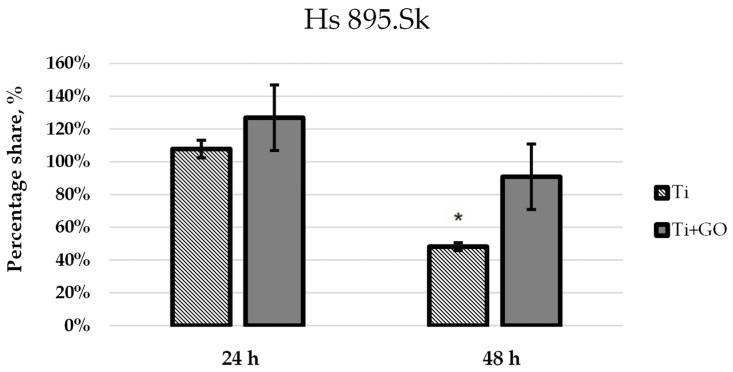
Percentage of viable Hs 895.Sk cells within the observed field after seeding on Ti and Ti + GO surfaces following 24 h and 48 h incubation, relative to the control set at 100%. Statistical analysis of the obtained results was carried out using ANOVA, (*—statistically significant result).

**Figure 11:**
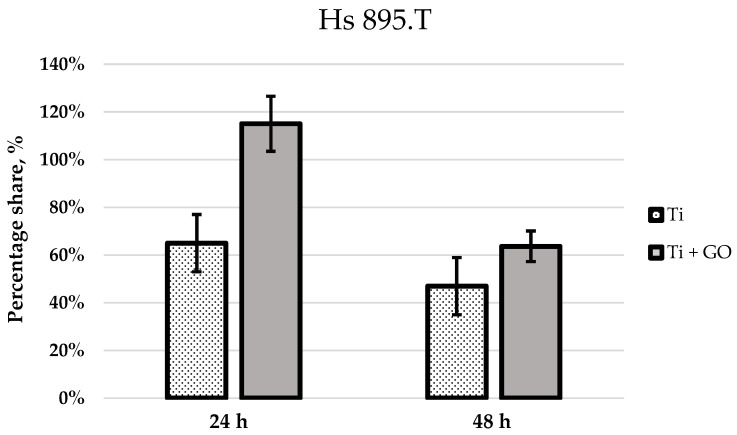
Percentage of viable Hs 895.T cells within the observed field after seeding on Ti and Ti + GO surfaces following 24 h and 48 h incubation, relative to the control set at 100%. Statistical analysis of the obtained results was carried out using ANOVA.

Based on the confocal images, the number of live cells within the analyzed field of view was quantified. Average values were calculated from three independent images for each experimental group and presented as bar graphs. Figure 10 shows the number of viable fibroblast cells (Hs 895.Sk) cultured on titanium foil and titanium foil with a graphene oxide layer after 24 and 48 h of incubation, relative to the control, defined as 100%. Figure 11 presents analogous results for melanoma cells (Hs 895.T).

Both the titanium foil (Ti) and the Ti + GO substrate exhibited a favorable effect on cell growth and proliferation. Only sporadic apoptotic cells were observed, mainly on the Ti surface (Figure 8f and Figure 9i,j). Importantly, no dead cells were detected on the graphene oxide-modified surface (Ti + GO), indicating a very high cytocompatibility of this functionalized structure.

Statistical analysis of the results (Figure 10 and Figure 11) revealed significant differences in the number of viable cells observed within the field of view, depending on the type of substrate used. After 24 h of culturing human skin fibroblasts Hs 895.Sk on Ti and Ti + GO surfaces, the number of cells was noticeably higher for Ti + GO, reaching approximately 125% of the control value, while on pure titanium, it was close to 105%. A similar trend was observed for cancerous Hs 895.T cells, where the proliferation of cells grown on Ti + GO reached nearly 120% of the control, compared to about 65% on Ti.

After 48 h of incubation, a decrease in the number of cells was observed for both cell lines on both surfaces, which may result from the limited growth area and cell confluence within the analyzed field of view, or from proliferation inhibition due to cell-to-cell contact. Nevertheless, even at this stage, cells on Ti + GO maintained higher viability compared to those on Ti. For Hs 895.Sk fibroblasts, these values were approximately 90% vs. 47%, and for Hs 895.T cells—about 65% vs. 48%. This indicates that the difference in cell number for both types of cell lines after 48 h of incubation was around 17%, in favor of the graphene oxide-modified substrate.

### 3.4. Genotoxicity Results

**Figure 12:**
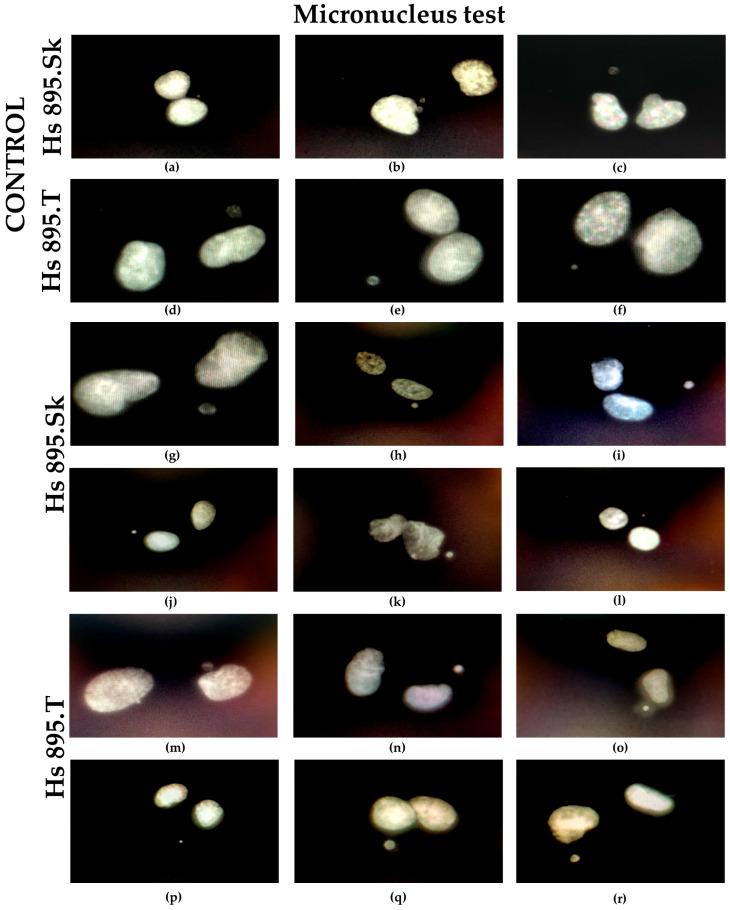
Results of the micronucleus test performed on healthy fibroblast and cancer cell lines: control samples for Hs 895.Sk (**a**–**c**) and Hs 895.T (**d**–**f**), as well as samples previously exposed to graphene oxide flakes for Hs 895.Sk (**g**–**l**) and Hs 895.T (**m**–**r**), visualized using a ZEISS Imager Z2 microscope equipped with a UV module and Metafer software (mag. 1000×).

Figure 12 presents the results of the micronucleus test performed on Hs 895.Sk and Hs 895.T cell lines exposed to graphene oxide (GO) flakes. Control images for human skin fibroblasts Hs 895.Sk (Figure 12a–c) and melanoma cells Hs 895.T (Figure 12d–f) are shown in comparison with cells exposed to GO (Hs 895.Sk—Figure 12g–l; Hs 895.T—Figure 12m–r). Microscopic analysis was conducted using a ZEISS Imager Z2 system equipped with a UV attachment and Metafer 3 software, enabling precise detection of micronuclei.

Quantitative results are summarized in Table 2, which includes the numbers of mononucleated and binucleated cells as well as the number of micronuclei recorded in control and GO-exposed cells. For both cell lines, the number of micronuclei in the test samples did not exceed that of the control samples. In the Hs 895.Sk control group, a total of 22 micronuclei were detected (20 in mononucleated cells and 2 in binucleated cells), while after GO exposure, 10 micronuclei were observed (9 and 1, respectively). Similarly, for the Hs 895.T line, the corresponding values were 2 (1 + 1) in the control and 2 (1 + 1) in the test group.

The absence of an increased percentage of micronucleated cells under GO exposure indicates that no elevated genetic instability or chromosomal breakage occurred, suggesting that graphene oxide does not induce genotoxic effects. Furthermore, no changes were observed in the ratio of mono- to binucleated cells in either cell line, confirming that graphene oxide does not interfere with cell division or the cell cycle. 

### 3.5. Morphology of Healthy Fibroblast Cells of Hs 895.Sk and Hs 895.T Lines

**Figure 13:**
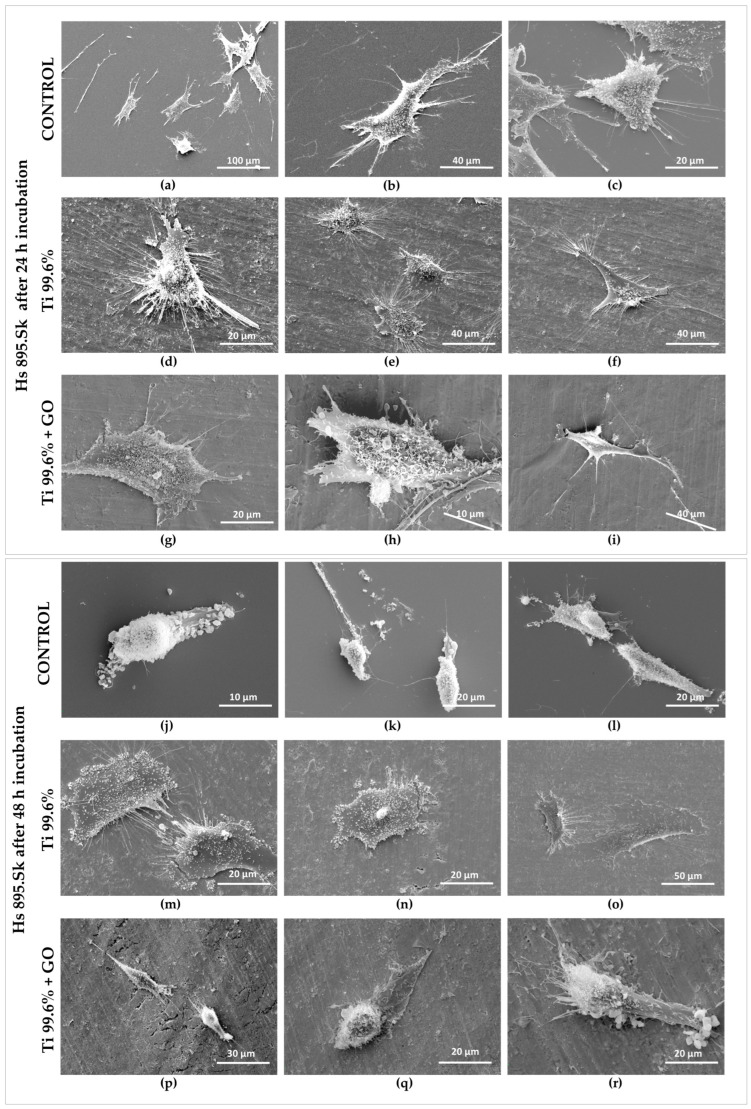
Morphology of healthy fibroblast cells of the Hs 895.Sk line cultured on a polystyrene plate (**a**–**c**), Ti substrate (**d**–**f**), and Ti + GO substrate (**g**–**i**) after 24 h of incubation, as well as fibroblasts of the Hs 895.Sk line cultured on a polystyrene plate (**j**–**l**), Ti substrate (**m**–**o**), and Ti + GO substrate (**p**–**r**) after 48 h of incubation, imaged using a Quanta 250 FEG scanning electron microscope.

**Figure 14:**
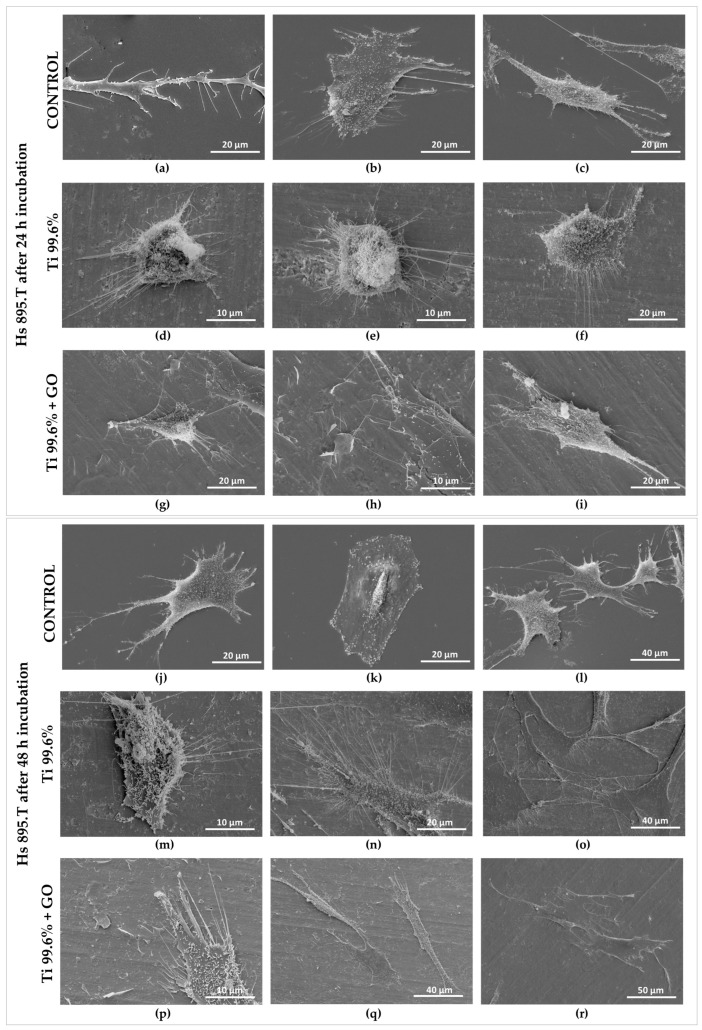
Morphology of melanoma cancer cells of the Hs 895.T line cultured on a polystyrene plate (**a**–**c**), Ti substrate (**d**–**f**), and Ti + GO substrate (**g**–**i**) after 24 h of incubation, as well as Hs 895.T cells cultured on a polystyrene plate (**j**–**l**), Ti substrate (**m**–**o**), and Ti + GO substrate (**p**–**r**) after 48 h of incubation, imaged using a Quanta 250 FEG scanning electron microscope.

The morphology of human skin fibroblasts of the Hs 895.Sk line (Figure 13a–r) and melanoma cells of the Hs 895.T line (Figure 14a–r), cultured on a polystyrene plate (Figure 13a–c,j–l and Figure 14a–c,j–l), titanium foil 99.6% (Ti) (Figure 13d–f,m–o and Figure 14d–f,m–o), and titanium foil coated with a graphene oxide layer (Ti + GO) (Figure 13g–i,p–r and Figure 14g–i,p–r), after 24 h (Figure 13a–i and Figure 14a–i) and 48 h of incubation (Figure 13j–r and Figure 14j–r), was evaluated using scanning electron microscopy (SEM).

Based on the obtained images (Figure 13 and Figure 14), it was found that Hs 895.Sk fibroblasts exhibited numerous cytoplasmic extensions and filopodia after just 24 h of culture, indicating active adhesion and good biological condition. After 48 h, further cell development was observed, confirmed by pronounced spreading and stable contact with the substrate, suggesting favorable growth conditions on all analyzed surfaces.

In the case of Hs 895.T melanoma cells, significant morphological differences depending on the substrate were observed after 24 h. Cells cultured on Ti foil showed a more spherical shape, which may indicate weaker adhesion to this surface. In contrast, on the Ti + GO surface, cells displayed more dispersed and flattened forms, characteristic of stable attachment. After 48 h of incubation, Hs 895.T cells on both Ti and Ti + GO exhibited an elongated shape and numerous protrusions and filopodia, confirming proper adhesion and proliferative activity.

### 3.6. Results of Bacterial Growth Inhibition Tests

**Figure 15:**
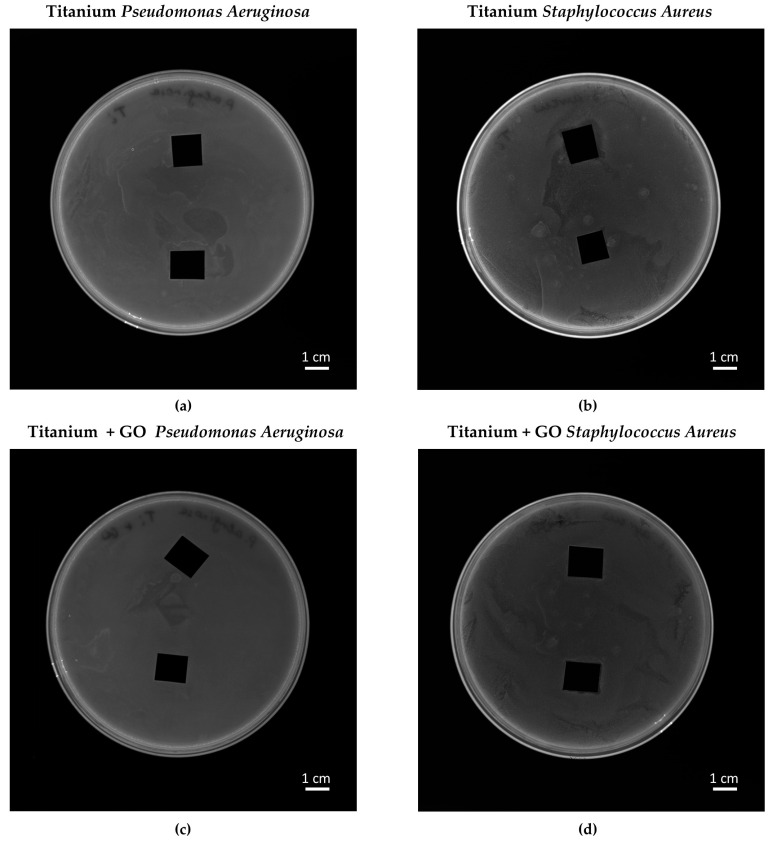
Inhibition zones of *Pseudomonas aeruginosa* (**a**,**c**) and *Staphylococcus aureus* (**b**,**d**) in contact with Ti (**a**,**b**) and Ti + GO (**c**,**d**) surfaces.

Figure 15 presents the results of the bacterial growth inhibition zone test for *Pseudomonas aeruginosa* and *Staphylococcus aureus* in contact with titanium samples (Ti) and titanium coated with graphene oxide (Ti + GO). Based on the obtained results (Figure 15), no distinct inhibition zones were observed around either the Ti or Ti + GO samples for the tested bacterial strains. This indicates that neither of the analyzed materials exhibited significant bactericidal or bacteriostatic properties under the conditions of the diffusion test performed.

**Figure 16:**
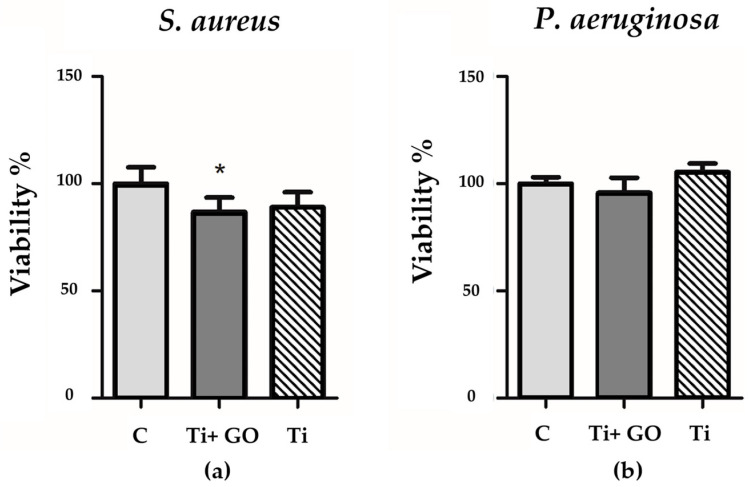
Viability of *Staphylococcus aureus* (**a**) and *Pseudomonas aeruginosa* (**b**) after contact with Ti and Ti + GO surfaces. Statistical analysis of the obtained results was carried out using ANOVA (*—statistically significant result).

Additionally, the viability of *Pseudomonas aeruginosa* and *Staphylococcus aureus* after exposure to Ti and Ti + GO surfaces was evaluated (Figure 16). The deposition of a GO layer on titanium resulted in a reduction in *Staphylococcus aureus* and *Pseudomonas aeruginosa* viability by approximately 10% and 4%, respectively, compared to Ti samples. This may suggest a slight antimicrobial activity associated with the graphene-based surface modification.

**Figure 17:**
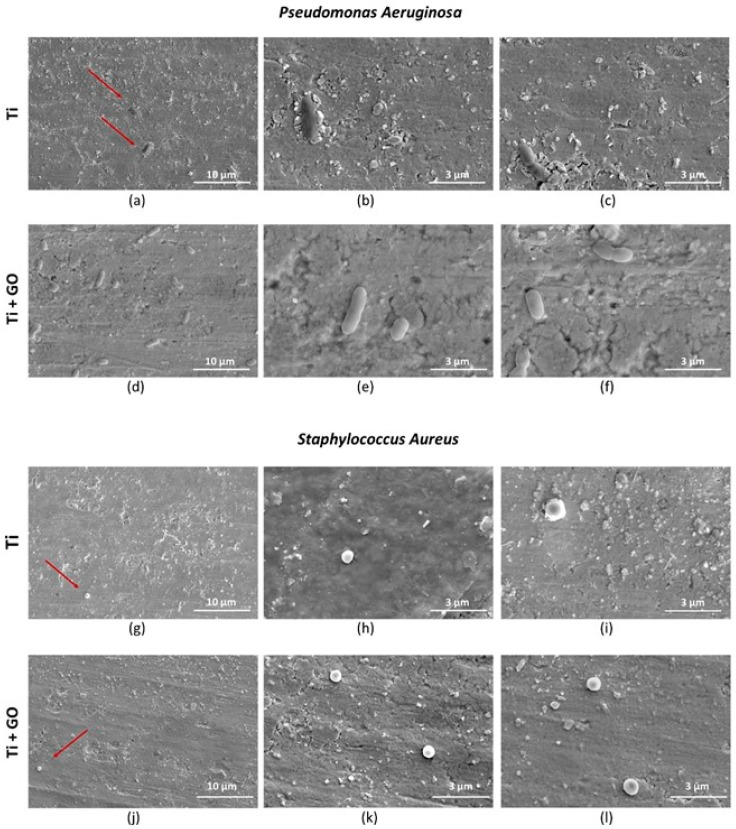
Morphology of *Pseudomonas aeruginosa* bacteria cultured on Ti (**a**–**c**) and Ti + GO (**d**–**f**), as well as morphology of *Staphylococcus aureus* bacteria cultured on Ti (**g**–**i**) and Ti + GO (**j**–**l**), imaged using a Quanta 250 FEG scanning electron microscope, arrows point to the bacteria.

Analysis of morphological changes in *Pseudomonas aeruginosa* and *Staphylococcus aureus* cells using scanning electron microscopy (Figure 17) revealed normal bacterial cell structures after contact with both types of substrates. The absence of deformation or morphological damage suggests that the antibacterial effect of the GO layer, although noticeable in the viability indicators, does not result from direct destruction of the microbial cell structure.

## 4. Discussion

The novelty of depositing a surface layer on titanium alloys, in contrast to the current state of knowledge, involves not only surface activation but also the possibility of embedding graphene nanostructures into the surface layer. This mechanism was confirmed in publication [27]. Such embedding is possible not only due to surface activation but also due to improved wettability.

To determine the effect of graphene oxide (GO) deposited on titanium foil, structural, corrosion, microbiological, biological, and genotoxicity studies were carried out. Structural surface analyses performed using scanning electron microscopy (SEM) and FTIR spectroscopy confirmed the presence of GO flakes deposited on the titanium foil surface. Distinct flake structures and boundaries of individual GO sheets were observed on Ti surfaces. The spectroscopic analysis revealed strong absorption bands corresponding to the stretching vibrations of hydroxyl (O–H) groups and aliphatic C–H bonds.

In the corrosion studies, it was observed that coating the titanium foil with graphene oxide limited the access of electrolytes and oxygen to the metal surface. The electrochemical potential of Ti + GO stabilized rapidly and remained constant over time. The higher OCP value (1.1774 V) compared to Ti indicates lower surface activity, which minimizes the risk of corrosion processes and provides better electrochemical properties. In the context of implant applications, this has a positive effect on mechanical stability and biocompatibility.

The results of biological tests clearly demonstrated that surface modification of titanium with GO promotes proper cellular behavior of human skin fibroblasts and may contribute to a reduction in microbial development. Analysis of fibroblast (Hs 895.Sk) viability and morphology showed that Ti + GO provided a more favorable microenvironment for cell growth than unmodified titanium. The cells exhibited distinct flattening and numerous cytoplasmic extensions, indicative of active adhesion and proper cytoskeletal function. These findings are consistent with literature reports suggesting that oxygen-containing functional groups present in GO enhance surface hydrophilicity and surface energy, facilitating the adsorption of adhesion proteins and promoting stable cell–substrate interactions [3,8,9].

Similar trends were observed for melanoma cells (Hs 895.T); however, their proliferation and morphological response differed from fibroblasts. After 24 h, the melanoma cells adhered more strongly to Ti + GO than to Ti, while after 48 h, they exhibited more pronounced spreading and the presence of filopodia on both substrates. Hs 895.T cells cultured on Ti + GO showed a more spread and adherent morphology; however, this represents structural adaptation to the substrate rather than evidence of changes in migratory potential. Since the present work did not include migration-focused tests, no conclusions can be drawn about GO-related modulation of cancer cell motility. Nonetheless, the absence of migration-specific assays does not preclude other GO-associated cellular effects described in previous studies. Some studies have shown that GO can modulate proliferation pathways and inhibit the growth of certain cancer cells through oxidative stress generation or modification of adhesion-related signaling pathways [10,11,12]. Ti + GO improves early adhesion and viability of both fibroblasts and Hs 895.T cells; this does not imply support of malignant proliferation. Additional long-term and in vivo investigations would be required to assess tumor implant interactions in oncologic patients. The results obtained here may be consistent with these findings, although further mechanistic investigation is required. The improved viability of cells on Ti–GO can be explained by the combined effect of GO’s surface chemistry and nanoscale morphology. The abundant oxygen-containing groups (–OH, –COOH, –C=O) increase hydrophilicity and surface free energy, promoting rapid adsorption of adhesion-related proteins from the culture medium, which enhances integrin-mediated attachment. Additionally, the negative surface charge of GO at physiological pH stabilizes the protein conditioning layer and facilitates the formation of a favorable cell–material interface. The wrinkled micro-/nanostructure of the GO coating provides multiple anchoring points for filopodia, supporting cell spreading and cytoskeletal organization, as confirmed by SEM. Together, these features likely contribute to the higher viability observed on Ti–GO compared with unmodified titanium. To further verify the biological safety of the coating, we next evaluated potential DNA damage and chromosomal instability using the micronucleus assay.

The micronucleus test results clearly confirmed the absence of genotoxic effects in both healthy fibroblasts and melanoma cells. The proportions of mono- and binucleated cells and the number of micronuclei remained similar to those of control samples, indicating preserved genoic stability and no induction of DNA damage upon exposure to Ti + GO. This is a crucial requirement for the clinical acceptance of biomaterials, especially in long-term tissue contact applications (ISO 10993 standard, International Organization for Standardization, Geneva, Switzerland) [40].

The in vitro effects observed for Ti + GO may be relevant for implant performance in vivo. Enhanced fibroblast adhesion is widely recognized as a prerequisite for early soft-tissue integration and long-term implant stability [41]. Studies have shown that GO-containing surfaces promote protein adsorption and fibroblast attachment in ways that support peri-implant tissue sealing [42]. The improved electrochemical stability and passive behavior of Ti + GO observed in our study are consistent with the literature, which links stable OCP and low corrosion susceptibility to enhanced implant durability under physiological conditions [43]. Finally, the absence of genotoxicity aligns with ISO 10993 requirements and indicates a low likelihood of initiating inflammatory or mutagenic processes in vivo [44]. The absence of genotoxic effects is especially relevant for chronic implant applications, where prolonged biomaterial exposure must not induce DNA damage or disrupt the local tissue environment.

Bacterial response analysis showed no inhibition zones in the agar diffusion test, which may result from limited nanomaterial diffusion and the necessity of direct contact between GO and bacterial cells. However, bacterial viability assays confirmed a slight but noticeable reduction in the number of viable *Staphylococcus aureus* and *Pseudomonas aeruginosa* cells in contact with Ti + GO, suggesting partial antibacterial activity of the modified surface. The mechanisms of GO’s antibacterial action may involve direct membrane damage, protein and lipid adsorption, generation of reactive oxygen species (ROS), and disruption of biofilm integrity [11,12,13,14]. As no visible morphological damage of bacterial structures was observed, the likely mechanism is ROS generation—commonly attributed to graphene-family materials [45]. Additionally, the physicochemical parameters of the applied material strongly affect its antibacterial efficacy. Given the combination of GO with titanium, another possible mechanism involves indirect isolation of bacterial cells from nutrients [46].

Overall, the obtained results suggest that GO may serve as a beneficial component for reducing bacterial infections on implant surfaces. Nevertheless, further optimization of the nanostructure and the amount of the deposited layer is necessary to enhance its antimicrobial effectiveness.

## 5. Conclusions

Graphene oxide (GO) flakes were uniformly deposited onto model implant samples made of titanium foil, as confirmed by imaging using scanning transmission electron microscopy (STEM).

Infrared surface analysis (FTIR) revealed characteristic absorption bands typical of graphene oxide, corresponding to hydroxyl (O–H) and aliphatic C–H stretching vibrations.

Deposition of GO nanostructures on the titanium surface reduced the access of corrosive electrolytes and oxygen to the metallic surface of the samples. The electrochemical potential curve stabilized rapidly and remained constant over time. The higher OCP value indicated lower corrosion activity, which positively influences the practical applicability of the material for implant manufacturing.

The presence of GO nanostructures on Ti improved the adhesion and viability of the tested cell lines, Hs 895.Sk and Hs 895.T, while reducing the number of dead or damaged cells.

Neither Ti nor Ti + GO surfaces exhibited significant antibacterial properties. The materials did not alter the morphology of *Staphylococcus aureus* or *Pseudomonas aeruginosa* cells, nor did they produce inhibition zones around the samples.

## Figures and Tables

**Table 1:** The kinetic data derived from potentiodynamic polarization curves.

Sample	Ecorr. (V)	jcorr. (×10^−8^ A/cm^2^)	Polarization Resistance (kΩ)	Corrosion Rate (mm/year)
Ti	−0.044407	1.5345	912.25	0.00053389
Ti/GO	−0.057959	5.3702	552.96	0.00018684

**Table 2:** Number of micronuclei in control and experimental samples for Hs 895.Sk and Hs 895.T cell lines.

Sample	Ecorr. (V)	jcorr. (×10^−8^ A/cm^2^)	Polarization Resistance (kΩ)	Corrosion Rate (mm/year)
Ti	−0.044 ± 0.006	1.53 ± 0.16	912 ± 61	(5.33 ± 0.5) × 10^−4^
Ti + GO	0.057 ± 0.006	5.37 ± 0.56	552 ± 106	(1.86 ± 0.2) × 10^−4^

## Data Availability

The original contributions presented in this study are included in the article. Further inquiries can be directed to the corresponding author.
